# Parental Type D Personality and Children’s Hyperactive Behaviors: The Mediating Role of Parent–Child Interactive Activities

**DOI:** 10.3390/ijerph16071116

**Published:** 2019-03-28

**Authors:** Guan-Hao He, Li Liu, Esben Strodl, Zeng-Liang Ruan, Hui Jiang, Jin Jing, Yu Jin, Wei-Qing Chen

**Affiliations:** 1 Department of Biostatistics and Epidemiology, School of Public Health, Sun Yat-sen University, Guangzhou 510080, China; heguanh@mail2.sysu.edu.cn (G.-H.H.); liuli9931@163.com (L.L.); ruanzliang@mail2.sysu.edu.cn (Z.-L.R.); jiangh43@163.com (H.J.); 2 School of Psychology and Counselling, Queensland University of Technology, Brisbane 4059, Queensland, Australia; e.strodl@qut.edu.au; 3 Department of Women and Child Health, School of Public Health, Sun Yat-sen University, Guangzhou 510080, China; jingjin@mail.sysu.edu.cn (J.J.); jinyu@mail.sysu.edu.cn (Y.J.); 4 Department of Information Management, Xinhua College of Sun Yat-Sen University, Guangzhou 510080, China

**Keywords:** hyperactive behaviors, parental type D personality, parent–child interactive activities, children, mediation

## Abstract

This study explored the associations between parental Type D personality (TDP), parent–child interactive activities, and children’s hyperactive behaviors. Moreover, the study examined whether parent–child interactive activities mediated the associations between parental TDP and children’s hyperactive behaviors. A cross-sectional survey was conducted among children from all kindergartens in a district of a southern city in China. Data on parental TDP, the frequency of parent–child interactive activities, children’s hyperactive behaviors, and socio-demographic characteristics were provided by 47,648 parent–child dyads. Multiple regression analysis was employed to assess the associations between parental TDP, parent–child interactive activities, and children’s hyperactive behaviors. Mediation analysis was applied to explore the mediating role of parent–child interactive activities on the associations between parental TDP and children’s hyperactive behaviors. Parental TDP was negatively associated with the frequency of parent–child interactive activities and positively associated with children’s hyperactive behaviors. The frequencies of parent–child interactive activities were negatively associated with children’s hyperactive behaviors. The frequency of parent–child interactive activities partially mediated the associations between parental TDP and children’s hyperactive behaviors. Future research may consider parental TDP and parent–child interactive activities as potential important predictors of hyperactive behaviors in children. Such research will help identify further targets for intervention to reduce hyperactive behaviors in children.

## 1. Introduction

Hyperactive behaviors is the primary clinical symptom of attention-deficit/hyperactivity disorder (ADHD) [1], as well as being an important clinical marker for the presence of other neurodevelopmental disorders in early childhood [2]. Previous research has found that hyperactive behaviors in childhood could increase the risk of later adverse events in life including poor academic achievement [3], unemployment and low earnings [4], and suicide [5]. Additionally, preschool hyperactive behaviors can also place long-term economic burden on the family and country [6]. While genetic and biological factors play a significant role in the etiology of hyperactive behaviors [7], empirical studies have shown that family-related factors, including family income, parenting styles, and the parent–child relationship can make a direct contribution to the expression of hyperactive behaviors in childhood [8,9,10].

The frequencies of positive mother–child interactive activities, such as praising the child, talking/playing with the child, laughing with the child, doing something special with the child, and playing sports, hobbies, or games with the child, have been reported to be related to children’s lower levels of hyperactive behaviors during preschool years [11]. A randomized controlled trial also showed that parent–child interaction training (an intervention program including conversation, story reading, and dramatic play with preschoolers) could reduce preschool children’s hyperactive behaviors 1 year after intervention [12]. Unfortunately, these studies did not indicate which interactive activities were associated with a reduction in children’s hyperactive behaviors. This limits our ability to develop more specific and effective measures to prevent children’s hyperactive behaviors. In addition, little is known about the predictors of the frequency of parent–child interactive activities. Such knowledge can help in the development of more targeted interventions.

Personality refers to the relatively enduring styles of thinking, feeling, and acting of a person [13]. Several previous studies indicated that parental Big Five personality scores (that is, scores on the five dimensions of personality of Neuroticism, Extraversion, Openness, Agreeableness, and Conscientiousness [13]) were associated with parents’ involvement in parent–child interactive activities. For example, Bornstein demonstrated that mothers with higher levels of extraversion and openness engaged in more symbolic play with their children [14]. Similarly Wan et al. showed that the frequency and quality of mother–child communication were higher among mother–child dyads with high extraversion and low neuroticism, compared with the mother–child dyads with low extraversion and high neuroticism [15]. These emerging findings indicate that parental personality traits may have an influence on parent–child interactive activities. However, it is important to extend this line of research in order to investigate whether other personality constructs may also be relevant to the frequency of parent–child interactive activities. Such research will help clarify the key personality constructs that may facilitate the parent–child interactive activities that are protective to the development of hyperactive behaviors of children.

While unfortunately few previous researchers have paid attention to the association between parental personality and children’s hyperactive behaviors, there is some evidence of the impact of parental personality on externalizing behaviors in children. For instance, Prinzie et al. found that high emotional stability, conscientiousness, and extraversion personality in parents were associated with their children’s lower externalizing problem behaviors (including hyperactive behaviors, poor regulation of impulses, noncompliance, and aggression towards peers) [16,17]. More research is therefore needed to examine the relationships between other parental personality constructs and more specific forms of externalizing behaviors in children such as hyperactive behaviors. Again, such research will help clarify the key personality constructs that may be protective or risk factors to the development of hyperactive behaviors in children.

Type D personality (TDP) is a stable personality construct that refers to the combination of two traits: Negative affectivity and social inhibition [18,19]. Negative affectivity denotes the tendency to experience negative emotions like dysphoria, anxiety, irritability, and a negative view of self. Social inhibition refers to experiencing discomfort in social interactions, reticence, lack of social poise, and unwillingness to express in social interactions to avoid confrontation. TDP has been reported to be associated with fewer healthy behaviors, poorer physical status, as well as a poorer mental health status [20,21]. However, to date there have been no published investigations into the associations between parental TDP, parent–child interactive activities, and children’s hyperactive behaviors. Examining whether such associations exist will extend the literature by identifying parental personality constructs, in addition to the Big Five, that may be important in the development of hyperactive behaviors in children. Furthermore, examining whether the frequency of parent–child interactive activities may mediate the relationship between parental TDP and children’s hyperactive behaviors will also provide evidence for possible mechanisms which may guide the development of future public health interventions. Given these aims, the hypotheses of the present study are as follows:

(1) Parental TDP will be associated with less frequent parent–child interactive activities.

(2) Lower frequencies of parent–child interactive activities will be related to higher levels of children’s hyperactive behaviors.

(3) Parental TDP will be associated with higher levels of children’s hyperactive behaviors.

(4) The frequency of parent–child interactive activities will mediate the relationships between parental TDP and children’s hyperactive behaviors.

## 2. Materials and Methods

### 2.1. Participants and Procedures

Participants of this study were recruited from the baseline survey of a Children Cohort Study in China which aimed to estimate the influence of early-life family and school environment on children’s neurobehavioral development. This Children Cohort Study was carried out in a district of a southern city in China. The education department of the government sent invitations to participate in the study to the parents of newly enrolled children in all kindergartens within the district. The research team provided the study’s questionnaires to all kindergartens, and then kindergarten teachers helped to give the questionnaires to the parents who agreed to participate. Parents and children in this study were surveyed from 2014 to 2017. The mother and father of a child were both asked to complete a scale about TDP, and one of the parents was required to answer an additional questionnaire concerning the socio-demographics of children and parents, the parent–child interactive activities, and the hyperactive behaviors of children. A total of 56,444 parent–child dyads were approached, and, after excluding those with incomplete data, a remaining 47,648 parent–child dyads (84.4% of 56,444) were included in the analyses. Written informed consent was obtained from each of the children’s parents. The study was approved by the Ethics Committee of the School of Public Health at Sun Yat-sen University (ethics clearance No.: 2015-016).

### 2.2. Measures

#### 2.2.1. Hyperactive Behaviors

Children’s hyperactive behaviors were assessed using the Conners’ Hyperactivity Index (HI), which is a subscale from Parent Rating Scale-Revised (CPRS-48) [22]. The parent version of Conners’ Hyperactivity Index is a well validated screening tool, which has been widely used in epidemiological studies to measure hyperactive behaviors in children aged between 3 and 17 years old [23,24]. This scale was translated into Chinese and has proven to have a good reliability and validity [25]. The Cronbach α coefficient for the HI was 0.83 in our sample. The HI consist of 10 items rated on a four-point scale (0 = not at all to 3 = very much). The items were summed and then divided by 10 to get a mean score and the range is 0 to 3.0, which was used as a continuous measure in the analysis. Higher scores reflected greater symptomatology. The 90th percent HI score for the child’s age and gender is commonly used as the cut-off for establishing hyperactive behaviors [25], and provided a dichotomous measure for the analyses in this study.

#### 2.2.2. Type D Personality

The Type D Personality Scale (DS14) was used to assess parental TDP. The DS14 is comprised of the two factors of negative affectivity (NA) and social inhibition (SI). Each factor involved 7 items rated on a 5-point scale (0 = false to 4 = true). The score ranges from 0 to 28 for both NA and SI. Respondents with a score ≥10 on both subscales were regarded as having TDP [18]. The Cronbach α coefficient was 0.83 for NA and 0.72 for SI in the original Chinese version [26]. In our sample, the Cronbach α coefficient for NA was 0.90 for mothers and 0.86 for fathers. The Cronbach α coefficient in this study for SI was 0.70 for mothers and 0.73 for fathers.

#### 2.2.3. Parent–Child Interactive Activities

One of the parents rated the frequencies of parent–child interactive activities for both parents and the child over two for different time periods: Before their child was 1 year old and when their child was 1 to 3 years old. The items related to the parent–child interactive activities were selected based on previous literatures [11,12,27] and Chinese culture. The parent–child interactive activities before 1 year old included the frequency of the following activities: Singing with the child, chatting with the child, playing with the child, and enjoying outdoor activities with the child. The parent–child interactive activities from 1 to 3 years old included the frequency of the following activities: Reading with the child, singing with the child, chatting with the child, playing with the child, joining family and friends’ gatherings with the child, and enjoying outdoor activities with the child. The frequencies of all parent–child interactive activities were rated on a five-point scale: Never, <1 time per week, 1–2 times per week, 3–6 times per week, and every day. The scores of these items were summed up and then divided by the number of items to get two interactive indexes: The interactive index before 1 year old and the interactive index from 1 to 3 years old.

#### 2.2.4. Covariates

The potential covariate variables included parents’ sociodemographic characteristics of age, educational level (≤12th grade, high school, undergraduate), marital status (married, single), and children’s sociodemographic characteristics of age, gender, and migrant status (“no” means that the child has registered permanent residence in the researched city, “yes” means that the child does not have registered permanent residence in the researched city). The covariate variables that were significant at *p* < 0.1 in univariate analyses or widely reported in the literatures were controlled in the multiple regression models.

### 2.3. Statistical Analysis

We conducted descriptive analyses on the socio-demographic characteristics of participants with means and standardized deviation for continuous variables and proportion for categorical variables. *T*-tests were used for the comparisons for continuous variables between different groups, and *χ*^2^ tests were performed for the comparisons for categorical variables between different groups. Multiple linear regression and logistic regression were respectively performed to evaluate the associations between parental TDP, parent–child interactive activities, and children’s hyperactive behaviors, after controlling for the covariate variables. Because some HI included zero values, the value of ‘1′ was added to the HI score for each child, and then the new score was transformed by natural logarithm (ln) to reduce skewness, when the dependent variable was the HI score in linear regression. The natural log-scaled partial coefficient of linear regression can be exponentiated to express the percentage changes of ‘HI score + 1′. For example, in Model 1 of Table 3, the 0.111 *β* coefficient of maternal TDP means that a mother with TDP can lead to a “e^0.111^−1 = 11.7%” higher in children’s HI score, compared to the mother without TDP. For more detailed information concerning the meaning of the coefficients after a log transformation of Hyperactivity Index, please refer to the text in the first page of the Supplementary Material.

The mediation effect of parent–child interactive activities on the association between parental TDP and children’s hyperactive behaviors was assessed with a series of hierarchical linear regressions or logistic regressions after adjusting for the covariate variables. According to Baron and Kenny [28], the mediation effect is demonstrated when the following criteria are met: (1) The main independent variable (maternal/paternal/combined parental TDP) is significantly related to the main dependent variable (hyperactive behaviors of children; see Model 1 in Table 3; the partial coefficient was denoted by c); (2) the independent variable (maternal/paternal/combined parental TDP) is significantly associated with the mediator variable (parent–child interactive activity in the multiple linear model; see Table 2 and Table S1; the partial coefficient was denoted by a); and (3) the mediator variable (parent–child interactive activity) is significantly related to the dependent variable (hyperactive behaviors of children) when the independent variable (maternal/paternal/combined parental TDP) is controlled (see Model 2 in Table 3 and Table S3; the partial coefficient was denoted by b). Then the indirect effect of the independent variable on the dependent variable through a mediator was evaluated as a × b (continuous outcome) or exp (a × b) (namely OR^IE^; dichotomous outcome), and the direct effect of X on Y was estimated as c’ or exp (c’) (namely OR^DE^; c’ referred to the partial coefficient of parental TDP to hyperactive behavior in Model 2).

In order to know how much of the effect of the independent variable on the dependent variable operates indirectly through the mediator, an effect size measure named ’the proportion of mediation’ is used in the current study. The proportion of mediation is interpreted as the proportion of the effect of the independent variable on the dependent variable that is mediated by mediator [29]. The proportion of mediation was calculated as follows [29,30]:

Continuous outcome: $\frac{a\times b}{c}$; dichotomous outcome: $\frac{{\mathrm{OR}}^{\mathrm{DE}}\times ({\mathrm{OR}}^{\mathrm{IE}}-1)}{{\mathrm{OR}}^{\mathrm{DE}}\times {\mathrm{OR}}^{\mathrm{IE}}-1}$.

All analyses were conducted with SAS 9.4 (SAS Institute Inc, Cary, NC, USA). All of the P-values were two-sided. Type I errors were set at 0.05.

## 3. Results

### 3.1. Sample Characteristics

Of the surveyed 47,648 children, 54.2% were boys and the mean age was 3.45 years old (*SD* = 0.39). Further, 55.6% of the mothers and 63.0% of the fathers had a bachelor degree or above, and the mean age was 31.09 years (*SD* = 4.05) for mothers and 33.42 years (*SD* = 4.71) for fathers. Other socio-demographic characteristics of the subjects are shown in Table 1.

Based on the recommended cut-off value for the DS14, 11.7% of the mothers and 9.4% of the fathers were classified as TDP. According to the recommended cut-off value (90th percent HI score for the child’s age and gender) for HI, 13.6% of the surveyed children were regarded as having hyperactive behaviors.

### 3.2. Associations between Parental TDP and Parent–Child Interactive Activities

Table 2 presents the results of associations between parental TDP and the parent–child interactive index after controlling for the covariate variables in the multiple linear regressions. Maternal TDP was significantly and negatively associated with the parent–child interactive index at 0 to 1 year (*β*, −0.228, 95% CI [−0.250, −0.206]). Apart from this, maternal TDP was also negatively related to the parent–child interactive index at 1 to 3 years (*β*, −0.227, 95% CI [−0.245, −0.208]). Similar associations were also observed between paternal TDP and the parent–child interactive index at 0 to 1 year (*β*, −0.192, 95% CI [−0.216, −0.168]), as well as the parent–child interactive index at 1 to 3 years (*β*, −0.184, 95% CI [−0.204, −0.163]). In order to explore the influence of maternal TDP and paternal TDP concurrently, maternal TDP and paternal TDP were combined into an extra variable. This variable included four combinations of the presence or absence of maternal and paternal TDP to produce four categories: (1) Non-TDP mother and non-TDP father, (2) TDP mother and non-TDP father, (3) non-TDP mother and TDP father, or (4) TDP mother and TDP father. The results of the analyses indicate that the frequency of parent–child interactive activities was significantly lower in the families where both parents had TDP or either parent had TDP than in families where neither parent had TDP (see Table 2). In addition, the significantly negative associations between parental TDP and each type of parent–child interactive activity were also found (see Table S1, Supplementary Material).

### 3.3. Associations between Parent–Child Interactive Activities and Children’s Hyperactive Behaviors

Table 2 displays the results for the regression analyses using both a continuous and a dichotomous measure of hyperactive behaviors. For the parent–child interactive activities at 0 to 1 year, an increasing level of parent–child interactive index was found to be significantly associated with decreased symptoms of hyperactive behaviors in children, after controlling for the covariate variables (*β*, −0.009, 95% CI [−0.012, −0.006]; percentage change in HI scores, −0.9%, 95% CI [−1.2%, −0.6%]). When the dichotomous measure of hyperactive behaviors was used, an increasing level on the parent–child interactive index was also found to be significantly associated with decreased risk of hyperactive behaviors in children (OR, 0.885, 95% CI [0.857, 0.915]).

For the parent–child interactive activities at 1 to 3 years, a similar result was found in that the parent–child interactive index was significantly negatively associated with the symptoms of children’s hyperactive behaviors, after controlling for the covariate variables (*β*, −0.037, 95% CI [−0.041, −0.034]; percentage change in HI scores, −3.6%, 95% CI [−4.0%, −3.3%]). The same associations were found when using a dichotomous measure of hyperactive behaviors (OR, 0.746, 95% CI [0.718, 0.775]). In addition, the significantly negative associations between most types of parent–child interactive activities and children’s hyperactive behaviors were also found (see Table S2, Supplemental Materials).

### 3.4. Association of Parental TDP with Children’s Hyperactive Behaviors and Mediating Role of Parent–Child Interactive Activities

After controlling for covariate variables, both maternal TDP (*β*, 0.111, 95% CI [0.104, 0.118]; percentage change in HI scores, 11.7%, 95% CI [11.0%, 12.5%]; OR, 2.288, 95% CI [2.136, 2.450]) and paternal TDP (*β*, 0.097, 95% CI [0.090, 0.105]; percentage change in HI scores, 10.2%, 95% CI [9.4%, 11.1%]; OR, 2.081, 95% CI [1.929, 2.245]) were significant associated with greater symptoms and risk of children’s hyperactive behaviors (Table 3 in Model 1). In addition, compared to children with a non-TDP mother and a non-TDP father, those with a TDP mother and a non-TDP father, those with a non-TDP mother and a TDP father, or those with a TDP mother and a TDP father had higher symptoms and risk of hyperactive behaviors. When the parent–child interactive index was added into Model 1, the magnitudes of the associations between parental TDP and children’s hyperactive behaviors attenuated (Table 3 in Model 2). The parent–child interactive index at 0 to 1 year significantly mediated the associations between maternal TDP and children’s hyperactive behaviors (proportion of mediation: 1.0% for continuous outcome, 3.4% for dichotomous outcome). Similar mediation effects of the parent–child interactive index at 0 to 1 year were also observed between paternal TDP and children’s hyperactive behaviors (proportion of mediation: 1.2% for continuous outcome, 3.6% for dichotomous outcome). Moreover, the parent–child interactive index at 0 to 1 year served as a significant mediator between combined parental TDP (TDP mother and non-TDP father, non-TDP mother and TDP father, TDP mother and TDP father) and children’s hyperactive behaviors. The proportions of the mediations were 0.7% (or 2.7%), 0.5% (or 1.9%), and 1.0% (or 3.7%), respectively.

In terms of the parent–child interactive activities at 1 to 3 years, the parent–child interactive index significantly mediated the association between maternal TDP and children’s hyperactive behaviors (proportion of mediation: 5.3% for continuous outcome, 9.7% for dichotomous outcome), as well as the association between paternal TDP and children’s hyperactive behaviors (proportion of mediation: 6.4% for continuous outcome, 9.2% for dichotomous outcome) (Table 3). Furthermore, mediation effects of the parent–child interactive index at 1 to 3 year were also observed between combined parental TDP (TDP mother and non-TDP father, non-TDP mother and TDP father, TDP mother and TDP father) and children’s hyperactive behaviors. The proportions of the mediations were 5.7% (or 9.1%), 4.5% (or 6.9%), and 7.0% (or 9.7%), respectively. Additionally, the mediation effects of many types of parent–child interactive activities were also found (see Table S3, Supplementary Material).

## 4. Discussion

The present study used the cross-sectional baseline information of a Children Cohort Study in China to explore the associations amongst parental TDP, parent–child interactive activities, and children’s hyperactive behaviors in a sample of Chinese children aged around three years old and their parents. After adjusting for the parental sociodemographic characteristics of age, educational level, and marital status, as well as the children’s sociodemographic characteristics of age, gender, and migrant status, maternal, paternal, and combined parental TDP were associated with less frequent parent–child interactive activities. Besides, lower frequencies of parent–child interactive activities were related to higher levels of hyperactive behaviors in their child. In addition, maternal TDP, paternal TDP, and combined parental TDP were associated with higher levels of hyperactive behaviors in the child. Moreover, mediation analysis indicated that the frequency of parent–child interactive activities at 0 to 1 year old (interactive index, singing with child, chatting with child, and playing with child) partially mediated the associations between maternal/paternal/combined parental TDP and children’s hyperactive behaviors. Furthermore, the frequency of parent–child interactive activities at 1 to 3 years old (interactive index, reading with child, singing with child, chatting with child, playing with child, joining family and joining friends’ gatherings with child, and outdoor activities with child) partially mediated the associations between maternal/paternal/combined parental TDP and children’s hyperactive behaviors.

Our findings of associations between parental TDP and parent–child interactive activities are consistent with our first hypothesis, as well as some previous studies. For example, Wan et al. demonstrated that mothers with the personality of high extraversion and low neuroticism are more likely to engage in frequent and high quality mother–child communication compared with mothers with low extraversion and high neuroticism [15]. Bornstein also reported that mothers with the personality of high extraversion and openness spent more time in symbolic play with their children [14]. Unfortunately, these studies only examined the impact of maternal personality on parent–child interactive activities but ignored the effect of paternal personality. As such our results build upon the findings from previous studies by confirming our hypothesis that mothers with TDP would have lower frequencies of parent–child interactive activities than mothers without TDP. However, our results also extend previous findings by identifying the same relationship between TDP and parent–child interactive activities in fathers.

Our findings of the associations between parent–child interactive activities and hyperactive behaviors in children are also consistent with our second hypothesis, as well as with previous studies. For instance, Nomaguchi demonstrated that the frequency of mother–child interactive activities was negatively associated with children’s hyperactive behaviors during preschool years [11]. In addition, Strayhorn et al. conducted a randomized control trial and found that parent–child interaction training (including conversation, story reading, and dramatic play with preschoolers) could reduce preschool children’s hyperactive behaviors 1 year after intervention [12]. Moreover, Maynard and Harding also found that the frequencies of engaging in joint family activities have a beneficial influence on adolescents’ psychological well-being (which comprised of 5 scales including hyperactivity) [31]. This suggests that the beneficial effect of the frequency of joint family activities may be broader than just upon reducing hyperactive behaviors symptomatology. Similarly, Sen found that a higher frequency of family dinners was negatively associated with adolescents’ problem behaviors [32]. The finding from this study was interesting given that the authors controlled the other family characteristics, such as parent–adolescent relationship and family income, indicating that there is an independent effect for the frequency of the joint family activity. Our study therefore builds upon these previous findings by showing that the frequency of parents singing with their child, chatting with their child, playing with their child, and enjoying outdoor activities with their child and the interactive index at 0 to 1 year, as well as the frequency of parents reading with their child, singing with their child, chatting with their child, playing with their child, joining family and friends’ gatherings with their child, and enjoying outdoor activities with their child and the interactive index at 1 to 3 years, were negatively associated with children’s hyperactive behaviors.

To the best of our knowledge, this is the first study that has reported an association between parental TDP and hyperactive behaviors among children. Consistent with our third hypothesis, our findings showed that having a mother with TDP was associated with a 11.7% higher score on their child’s HI score and 2.288 times higher probability of their child meeting criteria for having hyperactive behaviors than a mother without TDP. Having a father with TDP was related to a 10.2% higher score on their child’s HI score and 2.081 times higher probability of their child meeting criteria for having hyperactive behaviors than a father without TDP. In addition, compared with children having a non-TDP mother and a non-TDP father, having a TDP mother and a non-TDP father was associated with a 12.4% higher score on their child’s HI score and 2.245 times higher probability of their child meeting criteria for having hyperactive behaviors; having a non-TDP mother and a TDP father was associated with a 10.3% higher score on their child’s HI score and 1.918 times higher probability of their child meeting criteria for having hyperactive behaviors; having a TDP mother and a TDP father was associated with a 12.1% higher score on their child’s HI score and 2.593 times higher probability of their child meeting criteria for having hyperactive behaviors. These findings do support an emerging line of research showing an association between parental personality and externalizing behaviors in children [16,17]. More relevant to our study, a meta-analysis of Chinese studies demonstrated that the maternal personality trait of extraversion was a protective factor against the later development of ADHD in their offspring [33]. As such, our findings are consistent with previous findings showing a negative link between parental TDP and hyperactive behaviors in their children.

Another interesting consideration is how parental TDP influences children’s hyperactive behaviors. In this study, we hypothesized that parents with TDP engaged in less parent–child interactive activities, which in turn is associated with higher levels of their children’s hyperactive behaviors, and the results of our mediation analysis supported this hypothesis. The results of this study demonstrate that the frequency of parent–child interactive activities was a significant mediator between parental TDP and childhood hyperactive behaviors (with the proportion of mediation effects ranging from 0.2% to 9.7% across continuous and dichotomous models of hyperactive behaviors). These results suggest that up to 9.7% of the adverse effect of parental TDP on children’s hyperactive behaviors was mediated by the frequency of parent–child interactive activities. Though no previous study explored this issue, some researchers have reported the mediation role of parenting on the associations between parental personality and children’s behavioral problems. For example, Kochanska et al. reported that the effect of maternal negative emotionality on children’s behavioral problems was mediated by parenting style [34]. Prinzie et al. also indicated that parents with low scores on emotional stability were more likely to use overactive parenting (reflects parental irritability, anger, meanness, and frustration) which was subsequently associated with an increase in symptoms of externalizing problem behaviors in their children [16]. Such findings indicated that parent–child interactive activity might be a possible path from parental TDP to hyperactive behaviors, which was demonstrated by our study.

While our findings are similar to previous studies conducted in Western countries, there is a need to further understand the cultural factors that may be relevant to the associations identified. There is evidence that Chinese parents value children’s achievements more than American parents, while American parents value their children’s connectedness and individuality more than Chinese parents [35]. Different parenting values may lead to different parent–child interactions. For instance, McNeely and Barber reported that the frequency of talking/listening to children and engaging in activities with children was lower among Chinese parents than parents in Western countries [36]. These findings indicate that cultural factors may moderate the relationships identified in this study and need to be considered with cross-cultural comparisons.

In addition, the finding that the prevalence of TDP in this study (11.7% for mothers and 9.4% for fathers) is close to the prevalence from previous studies conducted with Chinese samples (15.9% in mainland China [26] and 16.0% in Taiwan [37]) gives confidence in the generalizability of our findings. However, interestingly the prevalence rates of TDP in all of these Chinese samples is lower than the TDP prevalence in Western countries (31.1% in Germany [38] and 39% in America [39]). It is possible that the different prevalence of TDP may be attributed to Chinese Confucian culture, which emphasizes the ideology of collectivism and encourages people to socialize with others. There is also epidemiological evidence that Chinese people generally report better interpersonal flexibility than Caucasian Americans [40]. In addition, Confucian culture also promotes the concept of the “golden mean” and advocates inner peace and happiness. Perhaps such cultural beliefs are related to epidemiological findings that the Chinese population has lower rates of depression, dysthymia, and anxiety than the American population [41]. The differences in culture between China and the Western countries may therefore result in different prevalence rates of TDP; however, this needs further investigation in future studies. In addition, there is a need for future studies to compare the strength of the associations between parental TDP and children’s hyperactive behaviors across different cultures.

While this study has a number of strengths, such as the very large sample size of over 45,000 participants, several limitations also ought to be considered. First, the information of parent–child interactive activities at different stages was retrospectively reported by parents, which might lead to recall bias. Second, we did not collect information about the duration and the quality of parent–child interactive activities. This prevented us from understanding the relationship between these aspects of parent–child interactive activities and children’s hyperactive behaviors. Third, we did not collect data on parental mental health other than TDP. Collecting this data would allow a more refined understanding of the relationship between parental TDP and children’s hyperactive behaviors. Fourth, this study used the baseline information of a Children Cohort Study and so all variables were assessed concurrently. Therefore, we could not determine the casual relation of the parental TDP and parent–child interactive activities with hyperactive behaviors. Longitudinal studies are particularly needed in order to help determine the direction of causality.

Given the findings of this study, there are a number of research areas that need further clarification. Since the prevalence of TDP and the parent–child interactive activities may vary across cultures [26,36,37,38,39], it is important to conduct cross-culture studies to explore the potential influence of culture on these associations. In addition, our findings demonstrate that parental TDP could influence parents’ engagement in interactive behaviors with their children, which could in turn influence children’s hyperactive behaviors. Given that TDP is a stable personality trait [18,19] and we are not aware of any current intervention program, it would be helpful to increase the frequency of parent–child interactive activities for parents with TDP to block their negative effect on children’s hyperactive behaviors. Moreover, parent–child interactive activities only partially mediated the relations between parental TDP and children’s hyperactive behaviors, and the effect is relatively small, which indicates that other possible pathways may exist and need to be further explored.

## 5. Conclusions

This is the first study to find evidence for the associations between parental TDP, the frequency of parent–child interactive activities, and hyperactive behaviors in young Chinese children. In addition, the study showed a partial mediating role of parent–child interactive activities in the associations between parental TDP and children’s hyperactive behaviors. Based upon these findings, researchers may consider including parental TDP and parent–child interactive activities as possible predictors of hyperactive behaviors in children across different cultural populations. Such research will help identify further targets for intervention to reduce hyperactive behaviors in children.

## Figures and Tables

**Table 1:** Socio-demographic characteristics of participants.

Variable	Total	Hyperactive Behaviors		
		No	Yes	*p* ^a^
Child Age, mean (*SD*)	3.45 (0.39)	3.46 (0.39)	3.40 (0.38)	<0.001
Gender, *n* (%)				0.013
Boy	25,823 (54.2)	22,409 (86.8)	3414 (13.2)	
Girl	21,825 (45.8)	18,769 (86.0)	3056 (14.0)	
Migrant Status, *n* (%)				<0.001
No	19,032 (39.9)	16,676 (87.6)	2356 (12.4)	
Yes	28,616 (60.1)	24,502 (85.6)	4114 (14.4)	
Marital Status, *n* (%)				<0.001
Married	46,186 (96.9)	39,961 (86.5)	6225 (13.5)	
Single	1462 (3.1)	1217 (83.2)	245 (16.8)	
Maternal Age, mean (SD)	31.09 (4.05)	31.25 (4.06)	30.12 (3.87)	<0.001
Maternal Education, *n* (%)				<0.001
≤12th Grade	7788 (16.4)	6558 (84.2)	1230 (15.8)	
≤High School	13,358 (28.0)	11,429 (85.6)	1929 (14.4)	
≥Undergraduate	26,502 (55.6)	23,191 (87.5)	3311 (12.5)	
Paternal Age, mean (*SD*)	33.42 (4.71)	33.57 (4.72)	32.45 (4.49)	<0.001
Paternal Education, *n* (%)				<0.001
≤12th Grade	6174 (13.0)	5125 (83.0)	1049 (17.0)	
≤High School	11,458 (24.0)	9867 (86.1)	1591 (13.9)	
≥Undergraduate	30,016 (63.0)	26,186 (87.2)	3830 (12.8)	

^a^*χ*^2^ tests were used for categorical variables, *t* tests were used for continuous variables.

**Table 2:** Associations among parental TDP, parent–child interactive activities, and children’s hyperactive behaviors.

Variables	Parent–Child Interactive Index at 0–1 Year, *β* (95% CI)	Parent–Child Interactive Index at 1–3 Years, *β* (95% CI)	Hyperactive Behaviors	
			Continuous, *β* (95% CI)	Dichotomous, OR (95% CI)
Maternal TDP				
No	Ref	Ref	Ref	Ref
Yes	−0.228 (−0.250, −0.206) ***	−0.227 (−0.245, −0.208) ***	0.111 (0.104, 0.118) ***	2.288 (2.136, 2.450) ***
Paternal TDP				
No	Ref	Ref	Ref	Ref
Yes	−0.192 (−0.216, −0.168) ***	−0.184 (−0.204, −0.163) ***	0.097 (0.090, 0.105) ***	2.081 (1.929, 2.245) ***
Combined parental TDP				
Non-TDP mother and non-TDP father	Ref	Ref	Ref	Ref
TDP mother and non-TDP father	−0.193 (−0.220, −0.165) ***	−0.217 (−0.241, −0.193) ***	0.117 (0.108, 0.126) ***	2.245 (2.056, 2.451) ***
Non-TDP mother and TDP father	−0.116 (−0.149, −0.083) ***	−0.140 (−0.169, −0.111) ***	0.098 (0.087, 0.108) ***	1.918 (1.718, 2.141) ***
TDP mother and TDP father	−0.292 (−0.325, −0.260) ***	−0.258 (−0.285, −0.230) ***	0.114 (0.104, 0.124) ***	2.593 (2.348, 2.863) ***
Parent–child index at 0–1 year			−0.009 (−0.012, −0.006) ***	0.885 (0.857, 0.915) ***
Parent–child index at 1–3 years			−0.037 (−0.041, −0.034) ***	0.746 (0.718, 0.775) ***

Adjusted for gender, child’s age, child’s migrant status, marital status, maternal age, maternal education, paternal age, and paternal education. *** *p* < 0.001. Abbreviations: TDP, type D personality; Ref, reference group.

**Table 3:** Mediation effect of parent–child interactive activities on the associations between parental TDP and children’s hyperactive behaviors.

Characteristics	Hyperactive Behaviors (Dependent Variable, Y, *β*/OR, 95% CI)		
	Model 1 X→Y	Model 2 X + M→Y	Proportion of Mediation
Y taken as a continuous variable			
Maternal TDP (Independent Variable, X)	0.111 (0.104, 0.118) ***	0.110 (0.103, 0.117) ***	1.0%
Interactive Index at 0–1 year (Mediator, M)		−0.005 (−0.008, −0.002) **	
Paternal TDP (Independent Variable, X)	0.097 (0.090, 0.105) ***	0.096 (0.088, 0.103) ***	1.2%
Interactive Index at 0–1 year (Mediator, M)		−0.006 (−0.009, −0.004) ***	
Combined parental TDP (Independent Variable, X)			
TDP mother and non-TDP father (D_1_)	0.117 (0.108, 0.126) ***	0.116 (0.108, 0.125) ***	0.7%
Non-TDP mother and TDP father (D_2_)	0.098 (0.087, 0.108) ***	0.097 (0.087, 0.108) ***	0.5%
TDP mother and TDP father (D_3_)	0.114 (0.104, 0.124) ***	0.113 (0.103, 0.123) ***	1.0%
Interactive Index at 0–1 year (Mediator, M)		−0.004 (−0.008, −0.001) **	
Maternal TDP (Independent Variable, X)	0.111 (0.104, 0.118) ***	0.104 (0.097, 0.110) ***	5.3%
Interactive Index at 1–3 years (Mediator, M)		−0.032 (−0.035, −0.029) ***	
Paternal TDP (Independent Variable, X)	0.097 (0.090, 0.105) ***	0.091 (0.083, 0.098) ***	6.4%
Interactive Index at 1–3 years (Mediator, M)		−0.034(−0.037, −0.031) ***	
Combined parental TDP (Independent Variable, X)			
TDP mother and non-TDP father (D_1_)	0.117 (0.108, 0.126) ***	0.111 (0.102, 0.119) ***	5.7%
Non-TDP mother and father TDP (D_2_)	0.098 (0.087, 0.108) ***	0.093 (0.083, 0.104) ***	4.5%
TDP mother and TDP father (D_3_)	0.114 (0.104, 0.124) ***	0.106 (0.096, 0.116) ***	7.0%
Interactive Index at 1–3 years (Mediator, M)		−0.031 (−0.034, −0.027) ***	
Y taken as a dichotomous variable			
Maternal TDP (Independent Variable, X)	2.288 (2.136, 2.450) ***	2.245 (2.095, 2.405) ***	3.4%
Interactive Index at 0–1 year (Mediator, M)		0.919 (0.889, 0.950) ***	
Paternal TDP (Independent Variable, X)	2.081 (1.929, 2.245) ***	2.043 (1.893, 2.204) ***	3.6%
Interactive Index at 0–1 year (Mediator, M)		0.905 (0.876, 0.936) ***	
Combined parental TDP (Independent Variable, X)			
TDP mother and non-TDP father (D_1_)	2.245 (2.056, 2.451) ***	2.212 (2.026, 2.416) ***	2.7%
Non-TDP mother and TDP father (D_2_)	1.918 (1.718, 2.141) ***	1.901 (1.703, 2.123) ***	1.9%
TDP mother and TDP father (D_3_)	2.593 (2.348, 2.863) ***	2.535 (2.295, 2.801) ***	3.7%
Interactive Index at 0–1 year (Mediator, M)		0.925 (0.894, 0.956) ***	
Maternal TDP (Independent Variable, X)	2.288 (2.136, 2.450) ***	2.168 (2.023, 2.324) ***	9.7%
Interactive Index at 1–3 years (Mediator, M)		0.781 (0.751, 0.812) ***	
Paternal TDP (Independent Variable, X)	2.081 (1.929, 2.245) ***	1.987 (1.841, 2.145) ***	9.2%
Interactive Index at 1–3 years (Mediator, M)		0.766 (0.737, 0.796) ***	
Combined parental TDP (Independent Variable, X)			
TDP mother and non-TDP father (D_1_)	2.245 (2.056, 2.451) ***	2.137 (1.956, 2.334) ***	9.1%
Non-TDP mother and TDP father (D_2_)	1.918 (1.718, 2.141) ***	1.859 (1.665, 2.077) ***	6.9%
TDP mother and TDP father (D_3_)	2.593 (2.348, 2.863) ***	2.445 (2.212, 2.702) ***	9.7%
Interactive Index at 1–3 years (Mediator, M)		0.788 (0.758, 0.819) ***	

Adjusted for gender, child’s age, child’s migrant status, marital status, maternal age, maternal education, paternal age, and paternal education. ** *p* < 0.01, *** *p* < 0.001. Abbreviations: TDP, type D personality.

## Supplementary Materials

The following are available online at https://www.mdpi.com/1660-4601/16/7/1116/s1, Table S1: Associations between parental TDP and parent-child interactive activities. Table S2: Associations of parental TDP and parent-child interactive activities with children’s hyperactive behaviors. Table S3: Mediation effect of parent-child interactive activities on the associations between parental TDP and children’s hyperactive behaviors.
