# Supplementary material for: Parental Type D Personality and Children’s Hyperactive Behaviors: The Mediating Role of Parent–Child Interactive Activities
Source: Int J Environ Res Public Health. 2019 Mar 28;16(7):1116. doi: 10.3390/ijerph16071116 (PMC6480101; doi:10.3390/ijerph16071116)
Supplement: Supplementary file 1 [file ijerph-16-01116-s001.pdf]

Supplementary Material

Parental Type D Personality and Children's Hyperactive Behaviors: The Mediating Role of Parent–Child Interactive Activities

Guan-Hao He, Li Liu, Esben Strodl, Zeng-Liang Ruan, Hui Jiang, Jin Jing, Yu Jin and Wei-Qing Chen

Regression coefficients after the dependent variable is log-transformed

Log transformation is a useful method to reduce the skewness of the dependent variable in linear regression[1], [2]. Since some HI included zero values, the value of '1' was added to HI score for each child, and then the new score was transformed by natural logarithm (ln) to reduce skewness, when the outcome was the HI score in linear regression. In this case, the partial regression coefficients were used to express the strengths of associations of parental TDP and parent-child interactive activities with children's hyperactive behaviors. Further, the natural log scaled partial coefficient of linear regression can be exponentiated to express the percentage changes of 'HI score + 1' when the scores of parent-child interactive behaviors add one point, or when the mothers/fathers with TDP was compared with mothers/fathers without TDP. For example, in model 1 of Table 3, the 0.111 β coefficient of maternal TDP means that mother with TDP can lead to a "$e^{0.111}$-1=11.7%" higher in children's HI score, compared to the mother without TDP. The calculation of percentage change from the log scaled partial coefficient is as follows:

(1) $\ln Y = \beta_0 + \beta_1 X_1 + \beta_2 X_2 + \ldots\ldots + \beta_k X_k$,

$\ln Y' = \beta_0 + \beta_1(X_1 + 1) + \beta_2 X_2 + \ldots\ldots + \beta_k X_k$;

(2) $\ln Y' - \ln Y = \beta_0 + \beta_1(X_1 + 1) + \beta_2 X_2 + \ldots\ldots + \beta_k X_k - (\beta_0 + \beta_1 X_1 + \beta_2 X_2 + \ldots\ldots + \beta_k X_k) = \beta_1$;

(3) $\ln(\frac{Y'}{Y}) = \beta_1$;

(4) $\frac{Y'}{Y} = e^{\beta_1}$;

(5) $\frac{Y'-Y}{Y} = e^{\beta_1} - 1$;

(6) Percentage change of $Y = e^{\beta_1} - 1$;

In addition, Table S2 presents the β coefficients and its corresponding "percentage changes" in the linear regression concerning associations of parental TDP and parent-child interactive activities with children's hyperactive behaviors. In this linear regression, parental TDP and parent-child interactive activities were independent variables, while the HI scores after logarithm transformation was dependent variable.

Table S1. Associations between parental TDP and parent-child interactive activities.

Variable	Parent-child interactive activities at 0 to 1 year			
	**Singing**	**Chatting**	**Playing**	**Outdoor Activity**
Maternal TDP				
No	Ref	Ref	Ref	Ref
Yes	-0.291(-0.323,-0.259) ***	-0.185 (-0.211,0.160)***	-0.281(-0.310,-0252)***	-0.155(-0.182,-0.128)***
Paternal TDP				
No	Ref	Ref	Ref	Ref
Yes	-0.233(-0.269,-0.198)***	-0.168(-0.197,-0.140)***	-0.236(-0.268,-0.204)***	-0.130(-0.160,-0.101)***
Combined parental TDP				
Non-TDP mother and non-TDP father	Ref	Ref	Ref	Ref
TDP mother TDP and non-TDP father	-0.268(-0.309,-0.227)***	-0.130(-0.163,-0.097)***	-0.237(-0.274,-0.200)***	-0.135(-0.170,-0.101)***
Non-TDP mother and TDP father	-0.158(-0.208,-0.109)***	-0.078(-0.118,-0.038)***	-0.141(-0.186,-0.097)***	-0.086(-0.127,-0.044)***
TDP mother and TDP father	-0.344(-0.392,-0.295)***	-0.273(-0.311,-0.234)***	-0.360(-0.404,-0.317)***	-0.193(-0.233,-0.153)***

Variable	Parent-child interactive activities at 1 to 3 years					
	**Reading**	**Singing**	**Chatting**	**Playing**	**Gatherings[a]**	**Outdoor Activity**
Maternal TDP						
No	Ref	Ref	Ref	Ref	Ref	Ref
Yes	-0.257(-0.287,-0.227)***	-0.278(-0.308,-0.249)***	-0.184(-0.206,-0.162)***	-0.275(-0.303,-0.248)***	-0.179(-0.206,-0.152)***	-0.186(-0.216,-0.155)***
Paternal TDP						
No	Ref	Ref	Ref	Ref	Ref	Ref
Yes	-0.224(-0.257,-0.191)***	-0.224(-0.256,-0.192)***	-0.167(-0.192,-0.143)***	-0.231(-0.262,-0.201)***	-0.133(-0.163,-0.104)***	-0.124(-0.157,-0.090)***
Combined parental TDP						
Non-TDP mother and non-TDP father	Ref	Ref	Ref	Ref	Ref	Ref
TDP mother TDP and non-TDP father	-0.240(-0.278,-0.202)***	-0.269(-0.306,-0.232)***	-0.120(-0.149,-0.092)***	-0.248(-0.283,-0.213)***	-0.230(-0.265,-0.196)***	-0.194(-0.233,-0.155)***
Non-TDP mother and TDP father	-0.178(-0.224,-0.133)***	-0.172(-0.217,-0.127)***	-0.065(-0.099,-0.030)***	-0.161(-0.204,-0.119)***	-0.174(-0.216,-0.133)***	-0.089(-0.135,-0.042)***
TDP mother and TDP father	-0.302(-0.347,-0.258)***	-0.313(-0.357,-0.269)***	-0.282(-0.315,-0.248)***	-0.334(-0.375,-0.293)***	-0.129(-0.169,-0.088)***	-0.185(-0.231,-0.140)***

Adjusted for gender, child's age, child's migrant status, marital status, maternal age, maternal education, paternal age, paternal education.

* $P<0.05$ ** $P<0.01$ *** $P<0.001$

[a] Gatherings refer to joining family and friends gatherings with child

Abbreviation: TDP: type D personality.

Table S2. Associations of parental TDP and parent-child interactive activities with children's hyperactive behaviors.

Variables	Hyperactive behaviors		
	Continuous Measure		Dichotomous Measure
	**β (95% CI)**	**Percentage change in score, % (95% CI)**	**OR (95% CI)**
Maternal TDP			
No	Ref	Ref	Ref
Yes	0.111(0.104,0.118)***	11.7 (11.0,12.5)***	2.288(2.136,2.450)***
Paternal TDP			
No	Ref	Ref	Ref
Yes	0.097(0.090,0.105)***	10.2 (9.4,11.1)***	2.081(1.929,2.245)***
Combined parental TDP			
Non-TDP mother and non-TDP father	Ref	Ref	Ref
TDP mother and non-TDP father	0.117(0.108,0.126)***	12.4(11.4,13.4)***	2.245(2.056,2.451)***
Non-TDP mother and TDP father	0.098(0.087,0.108)***	10.3(9.1,11.4)***	1.918(1.718,2.141)***
TDP mother and TDP father	0.114(0.104,0.124)***	12.1(11.0,13.2)***	2.593(2.348,2.863)***
Parent-child interactive activity at 0 to 1 year			
Singing	-0.002(-0.004,-0.001)*	-0.2 (-0.4,-0.1)*	0.953(0.931,0.975)***
Chatting	0.001(-0.002,0.003)	0.1 (-0.2,0.3)	0.946(0.921,0.973)***
Playing	-0.016(-0.018,-0.014)***	-1.6 (-1.8,-1.4)***	0.867(0.846,0.888)***
Outdoor Activity	-0.001(-0.003,0.001)	-0.1 (-0.3,0.1)	0.973(0.947,0.999)*
Interactive Index	-0.009(-0.012,-0.006)***	-0.9 (-1.2,-0.6)***	0.885(0.857,0.915)***
Parent-child interactive activity at 1 to 3 years			
Reading	-0.023(-0.025,-0.021)***	-2.3 (-2.5,-2.1)***	0.840 (0.820,0.861)***
Singing	-0.012(-0.014,-0.010)***	-1.2 (-1.4,-1.0)***	0.902 (0.880,0.925)***
Chatting	-0.004(-0.007,-0.002)**	-0.4 (-0.7,-0.2)**	0.899(0.872,0.927)***
Playing	-0.024(-0.026,-0.022)***	-2.4 (-2.6,-2.2)***	0.823 (0.803,0.845)***
Gatherings[a]	-0.034(-0.036,-0.032)***	-3.3 (-3.5,-3.1)***	0.809 (0.786,0.832)***
Outdoor Activity	-0.003(-0.005,-0.001)***	-0.3 (-0.5,-0.1)***	0.964(0.941,0.987)**
Interactive Index	-0.037(-0.041,-0.034)***	-3.6 (-4.0,-3.3)***	0.746(0.718,0.775)***

Adjusted for gender, child's age, child's migrant status, marital status, maternal age, maternal education, paternal age, paternal education.

* P<0.05 ** P<0.01 *** P<0.001

[a] Gatherings refer to joining family and friends gatherings with child.

Abbreviation: TDP: type D personality; Ref: reference group.

Table S3. Mediation effect of parent-child interactive activities on the associations between parental TDP and children's hyperactive behaviors.

Characteristics	Hyperactive behaviors (Dependent variable, Y, *β*/OR, 95% CI)		
	**Model 1** X→Y	**Model 2** X+M→Y	**Proportion of Mediation**
**Y taken as a continuous variable**			
**Parent-child interactive activities at 0-1 year**			
Maternal TDP (Independent Variable, X)	0.111(0.104,0.118)***	0.111(0.104,0.118)***	NS
Singing (Mediator, M)	---	0.000(-0.002,0.002)	
Maternal TDP (Independent Variable, X)	0.111(0.104,0.118)***	0.111(0.105,0.118)***	NS
Chatting (Mediator, M)	---	0.003(-0.001,0.005)	
Maternal TDP (Independent Variable, X)	0.111(0.104,0.118)***	0.107(0.100,0.114)***	3.3%
Playing (Mediator, M)	---	-0.013(-0.015,-0.011)***	
Maternal TDP (Independent Variable, X)	0.111(0.104,0.118)***	0.111(0.104,0.118)***	NS
Outdoor Activity (Mediator, M)	---	0.001(-0.002,0.003)	
Paternal TDP (Independent Variable, X)	0.097(0.090,0.105)***	0.097(0.089,0.104)***	NS
Singing (Mediator, M)	---	-0.001(-0.003,0.001)	
Paternal TDP (Independent Variable, X)	0.097(0.090,0.105)***	0.097(0.090,0.105)***	NS
Chatting (Mediator, M)	---	0.002(-0.000,0.005)	
Paternal TDP (Independent Variable, X)	0.097(0.090,0.105)***	0.094(0.086,0.101)***	3.4%
Playing (Mediator, M)	---	-0.014(-0.016,-0.012)***	
Paternal TDP (Independent Variable, X)	0.097(0.090,0.105)***	0.097(0.090,0.105)***	NS
Outdoor Activity (Mediator, M)	---	-0.000(-0.003,0.002)	
Combined parental TDP (Independent Variable, X)			
TDP mother and non-TDP father (D_1)	0.117(0.108,0.126)***	0.117(0.109,0.126)***	NS
Non-TDP mother and TDP father (D_2)	0.098(0.087,0.108)***	0.098(0.087,0.108)***	NS
TDP mother and TDP father (D_3)	0.114(0.104,0.124)***	0.114(0.104,0.124)***	NS
Singing (Mediator, M)		0.001(-0.001,0.002)	
Combined parental TDP (Independent Variable, X)			
TDP mother and non-TDP father (D_1)	0.117(0.108,0.126)***	0.117(0.109,0.126)***	0.3%
Non-TDP mother and TDP father (D_2)	0.098(0.087,0.108)***	0.098(0.087,0.108)***	0.2%
TDP mother and TDP father (D_3)	0.114(0.104,0.124)***	0.114(0.105,0.125)***	0.7%
Chatting (Mediator, M)		-0.003(-0.001,-0.006)**	
Combined parental TDP (Independent Variable, X)			
TDP mother and non-TDP father (D_1)	0.117(0.108,0.126)***	0.114(0.105,0.123)***	2.6%
Non-TDP mother and TDP father (D_2)	0.098(0.087,0.108)***	0.096(0.085,0.106)***	1.9%
TDP mother and TDP father (D_3)	0.114(0.104,0.124)***	0.109(0.099,0.120)***	4.1%
Playing (Mediator, M)		-0.013(-0.015,-0.011)***	
Combined parental TDP (Independent Variable, X)			
TDP mother and non-TDP father (D_1)	0.117(0.108,0.126)***	0.117(0.109,0.126)***	NS
Non-TDP mother and TDP father (D_2)	0.098(0.087,0.108)***	0.098(0.087,0.108)***	NS
TDP mother and TDP father (D_3)	0.114(0.104,0.124)***	0.114(0.104,0.124)***	NS
Outdoor Activity (Mediator, M)		0.001(-0.001,0.003)	

Table S3. *Cont.*

Characteristics	Hyperactive behaviors (Dependent variable, Y, β/OR, 95% CI)		
	Model 1 X→Y	Model 2 X+M→Y	Proportion of Mediation
**Parent-child interactive activities at 1-3 year**			
Maternal TDP (Independent Variable, X)	0.111(0.104,0.118)***	0.106(0.099,0.112)***	4.6%
Reading (Mediator, M)	---	-0.020(-0.022,-0.018)***	
Maternal TDP (Independent Variable, X)	0.111(0.104,0.118)***	0.108(0.101,0.115)***	2.3%
Singing (Mediator, M)	---	-0.009(-0.011,-0.007)***	
Maternal TDP (Independent Variable, X)	0.111(0.104,0.118)***	0.111(0.104,0.117)***	NS
Chatting (Mediator, M)	---	-0.001(-0.004,0.002)	
Maternal TDP (Independent Variable, X)	0.111(0.104,0.118)***	0.105(0.098,0.112)***	5.2%
Playing (Mediator, M)	---	-0.021(-0.023,-0.018)***	
Maternal TDP (Independent Variable, X)	0.111(0.104,0.118)***	0.105(0.098,0.112)***	5.2%
Gatherings[a] (Mediator, M)	---	-0.032(-0.034,-0.030)***	
Maternal TDP (Independent Variable, X)	0.111(0.104,0.118)***	0.111(0.104,0.117)***	NS
Outdoor Activity (Mediator, M)	---	-0.002(-0.004,0.000)	
Paternal TDP (Independent Variable, X)	0.097(0.090,0.105)***	0.092(0.085,0.100)***	4.9%
Reading (Mediator, M)	---	-0.021(-0.023,-0.019)***	
Paternal TDP (Independent Variable, X)	0.097(0.090,0.105)***	0.095(0.087,0.102)***	2.3%
Singing (Mediator, M)	---	-0.010(-0.012,-0.008)***	
Paternal TDP (Independent Variable, X)	0.097(0.090,0.105)***	0.097(0.089,0.104)***	NS
Chatting (Mediator, M)	---	-0.002(-0.005,0.001)	
Paternal TDP (Independent Variable, X)	0.097(0.090,0.105)***	0.092(0.085,0.100)***	5.2%
Playing (Mediator, M)	---	-0.022(-0.024,-0.020)***	
Paternal TDP (Independent Variable, X)	0.097 (0.090,0.105)***	0.093(0.085,0.100)****	4.5%
Gatherings[a] (Mediator, M)	---	-0.033(-0.035,-0.031)***	
Paternal TDP (Independent Variable, X)	0.097 (0.090,0.105)***	0.097(0.089,0.104)***	0.4%
Outdoor Activity (Mediator, M)	---	-0.003(-0.005,-0.001)*	
Combined parental TDP (Independent Variable, X)			
TDP mother and non-TDP father (D_1)	0.117(0.108,0.126)***	0.112(0.104,0.121)***	4.1%
Non-TDP mother and TDP father (D_2)	0.098(0.087,0.108)***	0.094(0.084,0.104)***	3.6%
TDP mother and TDP father (D_3)	0.114(0.104,0.124)***	0.108(0.098,0.118)***	5.3%
Reading (Mediator, M)		-0.020(-0.022,-0.018)***	
Combined parental TDP (Independent Variable, X)			
TDP mother and non-TDP father (D_1)	0.117(0.108,0.126)***	0.115(0.106,0.124)***	1.8%
Non-TDP mother and TDP father (D_2)	0.098(0.087,0.108)***	0.096(0.086,0.107)***	1.4%
TDP mother and TDP father (D_3)	0.114(0.104,0.124)***	0.111(0.101,0.122)***	2.2%
Singing (Mediator, M)		-0.008(-0.010,-0.006)***	

Table S3. *Cont.*

Characteristics	Hyperactive behaviors (Dependent variable, Y, *β*/OR, 95% CI)		
	**Model 1 X→Y**	**Model 2 X+M→Y**	**Proportion of Mediation**
Combined parental TDP (Independent Variable, X)			
TDP mother and non-TDP father (D_1)	0.117(0.108,0.126)***	0.117(0.108,0.126)***	NS
Non-TDP mother and TDP father (D_2)	0.098(0.087,0.108)***	0.098(0.087,0.108)***	NS
TDP mother and TDP father (D_3)	0.114(0.104,0.124)***	0.114(0.104,0.124)***	NS
Chatting (Mediator, M)		-0.001(-0.003,0.002)	
Combined parental TDP (Independent Variable, X)			
TDP mother and non-TDP father (D_1)	0.117(0.108,0.126)***	0.112(0.104,0.121)***	4.2%
Non-TDP mother and TDP father (D_2)	0.098(0.087,0.108)***	0.094(0.084,0.105)***	3.3%
TDP mother and TDP father (D_3)	0.114(0.104,0.124)***	0.107(0.097,0.118)***	5.9%
Playing (Mediator, M)		-0.020(-0.022,-0.018)***	
Combined parental TDP (Independent Variable, X)			
TDP mother and non-TDP father (D_1)	0.117(0.108,0.126)***	0.110(0.101,0.119)***	6.1%
Non-TDP mother and TDP father (D_2)	0.098(0.087,0.108)***	0.092(0.082,0.102)***	5.5%
TDP mother and TDP father (D_3)	0.114(0.104,0.124)***	0.110(0.100,0.120)***	3.5%
Gatherings[a] (Mediator, M)		-0.031(-0.034,-0.029)***	
Combined parental TDP (Independent Variable, X)			
TDP mother and non-TDP father (D_1)	0.117(0.108,0.126)***	0.117(0.108,0.126)***	NS
Non-TDP mother and TDP father (D_2)	0.098(0.087,0.108)***	0.097(0.087,0.108)***	NS
TDP mother and TDP father (D_3)	0.114(0.104,0.124)***	0.114(0.104,0.124)***	NS
Outdoor Activity (Mediator, M)		-0.001(-0.003,0.001)	NS
**Y taken as a dichotomous variable**			
**Parent-child interactive activities at 0-1 year**			
Maternal TDP (Independent Variable, X)	2.288(2.136,2.450)***	2.271(2.119,2.433)***	1.3%
Singing (Mediator, M)	---	0.974 (0.952,0.997)*	
Maternal TDP (Independent Variable, X)	2.288(2.136,2.450)***	2.274(2.122,2.436)***	1.1%
Chatting (Mediator, M)	---	0.967 (0.941,0.995)*	
Maternal TDP (Independent Variable, X)	2.288(2.136,2.450)***	2.215(2.067,2.373)***	5.8%
Playing (Mediator, M)	---	0.888(0.867,0.911)***	
Maternal TDP (Independent Variable, X)	2.288(2.136,2.450)***	2.284(2.132,2.446)***	NS
Outdoor Activity (Mediator, M)	---	0.989(0.963,1.017)	
Paternal TDP (Independent Variable, X)	2.081(1.929,2.245)***	2.064(1.913,2.227)***	1.6%
Singing (Mediator, M)	---	0.965(0.943,0.988)**	
Paternal TDP (Independent Variable, X)	2.081(1.929,2.245)***	2.067(1.915,2.230)***	1.3%
Chatting (Mediator, M)	---	0.960(0.933,0.987)**	
Paternal TDP (Independent Variable, X)	2.081(1.929,2.245)***	2.022(1.874,2.182)***	5.7%
Playing (Mediator, M)	---	0.880(0.859,0.902)***	
Paternal TDP (Independent Variable, X)	2.081(1.929,2.245)***	2.076(1.924,2.240)***	NS
Outdoor Activity (Mediator, M)	---	0.982(0.956,1.009)	

Table S3. *Cont.*

Characteristics	Hyperactive behaviors (Dependent variable, Y, β/OR, 95% CI)		
	Model 1<br>X→Y	Model 2<br>X+M→Y	Proportion of Mediation
Combined parental TDP (Independent Variable, X)			
TDP mother and non-TDP father (D_1)	2.245(2.056,2.451)***	2.232(2.044,2.438)***	NS
Non-TDP mother and TDP father (D_2)	1.918(1.718,2.141)***	1.911(1.712,2.134)***	NS
TDP mother and TDP father (D_3)	2.593(2.348,2.863)***	2.574(2.330,2.843)***	NS
Singing (Mediator, M)		0.978(0.956,1.001)	
Combined parental TDP (Independent Variable, X)			
TDP mother and non-TDP father (D_1)	2.245(2.056,2.451)***	2.236(2.048,2.442)***	0.7%
Non-TDP mother and TDP father (D_2)	1.918(1.718,2.141)***	1.914(1.714,2.136)***	0.5%
TDP mother and TDP father (D_3)	2.593(2.348,2.863)***	2.572(2.328,2.841)***	1.3%
Chatting (Mediator, M)		0.971(0.944,0.998)*	
Combined parental TDP (Independent Variable, X)			
TDP mother and non-TDP father (D_1)	2.245(2.056,2.451)***	2.186(2.002,2.388)***	4.8%
Non-TDP mother and TDP father (D_2)	1.918(1.718,2.141)***	1.889(1.692,2.109)***	3.3%
TDP mother and TDP father (D_3)	2.593(2.348,2.863)***	2.492(2.256,2.754)***	6.5%
Playing (Mediator, M)		0.892(0.871,0.914)***	
Combined parental TDP (Independent Variable, X)			
TDP mother and non-TDP father (D_1)	2.245(2.056,2.451)***	2.243(2.054,2.449)***	NS
Non-TDP mother and TDP father (D_2)	1.918(1.718,2.141)***	1.916(1.717,2.140)***	NS
TDP mother and TDP father (D_3)	2.593(2.348,2.863)***	2.589(2.344,2.860)***	NS
Outdoor Activity (Mediator, M)		0.992(0.966,1.020)	
**Parent-child interactive activities at 1-3 year**			
Maternal TDP (Independent Variable, X)	2.288(2.136,2.450)***	2.205(2.058,2.363)***	6.8%
Reading (Mediator, M)	---	0.859(0.838,0.880)***	
Maternal TDP (Independent Variable, X)	2.288(2.136,2.450)***	2.241(2.091,2.401)***	3.8%
Singing (Mediator, M)	---	0.925(0.902,0.948)***	
Maternal TDP (Independent Variable, X)	2.288(2.136,2.450)***	2.255(2.105,2.416)***	2.6%
Chatting (Mediator, M)	---	0.924(0.896,0.954)***	
Maternal TDP (Independent Variable, X)	2.288(2.136,2.450)***	2.189(2.043,2.346)***	8.1%
Playing (Mediator, M)	---	0.844(0.823,0.866)***	
Maternal TDP (Independent Variable, X)	2.288(2.136,2.450)***	2.219(2.071,2.377)***	6.0%
Gatherings[a] (Mediator, M)	---	0.824(0.801,0.849)***	
Maternal TDP (Independent Variable, X)	2.288(2.136,2.450)***	2.279(2.127,2.441)***	NS
Outdoor Activity (Mediator, M)	---	0.980(0.956,1.004)	
Paternal TDP (Independent Variable, X)	2.081(1.929,2.245)***	2.012(1.865,2.172)***	6.8%
Reading (Mediator, M)	---	0.852(0.831,0.873)***	
Paternal TDP (Independent Variable, X)	2.081(1.929,2.245)***	2.042(1.892,2.203)***	3.8%
Singing (Mediator, M)	---	0.915(0.893,0.938)***	
Paternal TDP (Independent Variable, X)	2.081(1.929,2.245)***	2.050(1.900,2.212)***	2.8%
Chatting (Mediator, M)	---	0.916(0.887,0.945)***	
Paternal TDP (Independent Variable, X)	2.081(1.929,2.245)***	2.001(1.854,2.160)***	7.8%
Playing (Mediator, M)	---	0.836(0.815,0.858)***	

Table S3. *Cont.*

Characteristics	Hyperactive behaviors (Dependent variable, Y, β/OR, 95% CI)		
	Model 1<br>X→Y	Model 2<br>X+M→Y	Proportion of Mediation
Paternal TDP (Independent Variable, X)	2.081(1.929,2.245)***	2.034(1.885,2.195)***	5.1%
Gatherings[a] (Mediator, M)	---	0.817(0.793,0.841)***	
Paternal TDP (Independent Variable, X)	2.081(1.929,2.245)***	2.073(1.922,2.237)***	0.7%
Outdoor Activity (Mediator, M)	---	0.971(0.948,0.994)*	
Combined parental TDP (Independent Variable, X)			
TDP mother and non-TDP father (D_1)	2.245(2.056,2.451)***	2.172(1.988,2.372)***	6.3%
Non-TDP mother and TDP father (D_2)	1.918(1.718,2.141)***	1.872(1.676,2.090)***	5.4%
TDP mother and TDP father (D_3)	2.593(2.348,2.863)***	2.486(2.250,2.747)***	7.1%
Reading (Mediator, M)		0.863(0.842,0.885)***	
Combined parental TDP (Independent Variable, X)			
TDP mother and non-TDP father (D_1)	2.245(2.056,2.451)***	2.203(2.018,2.406)***	3.6%
Non-TDP mother and TDP father (D_2)	1.918(1.718,2.141)***	1.894(1.697,2.115)***	2.6%
TDP mother and TDP father (D_3)	2.593(2.348,2.863)***	2.536(2.296,2.802)***	3.7%
Singing (Mediator, M)		0.929(0.906,0.953)***	
Combined parental TDP (Independent Variable, X)			
TDP mother and non-TDP father (D_1)	2.245(2.056,2.451)***	2.225(2.038,2.430)***	1.6%
Non-TDP mother and TDP father (D_2)	1.918(1.718,2.141)***	1.909(1.710,2.131)***	1.0%
TDP mother and TDP father (D_3)	2.593(2.348,2.863)***	2.539(2.298,2.805)***	3.4%
Chatting (Mediator, M)		0.928(0.899,0.958)***	
Combined parental TDP (Independent Variable, X)			
TDP mother and non-TDP father (D_1)	2.245(2.056,2.451)***	2.160(1.978,2.359)***	7.2%
Non-TDP mother and TDP father (D_2)	1.918(1.718,2.141)***	1.871(1.676,2.090)***	5.4%
TDP mother and TDP father (D_3)	2.593(2.348,2.863)***	2.461(2.227,2.720)***	8.7%
Playing (Mediator, M)		0.849(0.827,0.871)***	
Combined parental TDP (Independent Variable, X)			
TDP mother and non-TDP father (D_1)	2.245(2.056,2.451)***	2.160(1.977,2.359)***	7.6%
Non-TDP mother and TDP father (D_2)	1.918(1.718,2.141)***	1.864(1.669,2.082)***	6.7%
TDP mother and TDP father (D_3)	2.593(2.348,2.863)***	2.540(2.299,2.806)***	3.9%
Gatherings[a] (Mediator, M)		0.828(0.804,0.852)***	
Combined parental TDP (Independent Variable, X)			
TDP mother and non-TDP father (D_1)	2.245(2.056,2.451)***	2.237(2.049,2.442)***	NS
Non-TDP mother and TDP father (D_2)	1.918(1.718,2.141)***	1.915(1.715,2.138)***	NS
TDP mother and TDP father (D_3)	2.593(2.348,2.863)***	2.584(2.339,2.854)***	NS
Outdoor Activity (Mediator, M)		0.982(0.958,1.006)	

Adjusted for gender, child's age, child's migrant status, marital status, maternal age, maternal education, paternal age, paternal education.

* $P<0.05$ ** $P<0.01$ *** $P<0.001$

0.000: The value was greater than 0 and less than 0.001, -0.000: The value was greater than -0.001and less than 0.

[a] Gatherings refer to joining family and friends gatherings with child.

NS: not significant (the mediation effect is not significant, so the proportion of mediation is not shown).
